# Comparative toxicity of urban wastewater and rainfall overflow in caged freshwater mussel *Elliptio complanata*

**DOI:** 10.3389/fphys.2023.1233659

**Published:** 2023-08-10

**Authors:** C. André, S. V. Duy, S. Sauvé, F. Gagné

**Affiliations:** ^1^ Aquatic Contaminants Research Division, Environment and Climate Change Canada, Montréal, QC, Canada; ^2^ Chemistry Department, Montreal University, Montréal, QC, Canada

**Keywords:** urban pollution, climate change, vitellogenin, oxidative stress, inflammation

## Abstract

Municipal effluents are well-recognized as disrupting sexual differentiation and reproduction in mussels. However, the contribution to this problem made by rainfall combined with sewer overflow (increased by rain due to climate change) is not well understood. The purpose of this study was to compare the neuroendocrine effects of municipal discharge and rainfall overflow on caged endemic mussel *Elliptio complanata*. To this end, mussels were experimentally caged and placed for 3 months at a municipal effluent dispersion plume site and at overflow sites. Data revealed that downstream surface water contained some pharmaceuticals (caffeine and carbamazepine) and accumulated significant levels of heterotrophic bacteria, but these effects were not observed at the overflow sites. The principal effects observed at the downstream site were increased soft tissue mass (and gonad index), inflammation, and Vtg proteins in male mussels as determined by a novel immunostaining methodology. The rainfall overflow sites had no effects on these markers, but were specifically associated with reduced Vtg proteins in females, dopamine (Dop), gonad lipids, and DNA strand breaks, with increased metallothioneins. In conclusion, the observed feminizing effects of municipal effluent were not additionally observed in mussels caged at rainfall overflow sites, although the latter exhibited a different pattern of toxicity.

## Introduction

Benthic invertebrates represent a group at risk from various pollutants, since they thrive at the sediment/water interface, where many contaminants are found. Bivalves are sessile and some live for many years, placing them at particular risk from pollution given that they filter-feed on suspended matter in surface water that may be affected by local sources of pollution, such as municipal and agricultural discharges. Moreover, such runoffs are increased by heavy rainfall events caused by global warming (Wang et al., 2023). Indeed, the global increase in temperature will bring about an increasing number of heavy rainfall events (Min et al., 2011). Wastewater treatment plants have limited capacity to handle large volumes of rainfall, and excess volumes (overflows) are mixed with incoming untreated wastewater and directly released into a receiving lake or river. Although these events last for relatively short periods of time, from hours to a few days, the continuous release of rainfall draining off from pavements and soil in urban areas, combined with raw wastewater, could pose a risk to aquatic organisms, especially resident organisms such as bivalves and other invertebrates. The monitoring of sites that are under the influence of rainfall overflows and municipal discharges would be useful in order to understand these effects in urbanized areas and help improve water management decisions.

The unionid *Elliptio complanata* is one of the most abundant freshwater species in the St. Lawrence River watershed (Downing and Downing, 1992). This makes this species a representative member of the invertebrate community for investigation of the cumulative impact of wastewater in the context of climate change. Members of this species can live for up to 10–15 years (Haag and Rypel, 2011; André et al., 2021). This mussel species is mainly dioecious, with intersex conditions occurring at a rate of less than 1% (Pipe, 1987). Synthesis of the egg yolk protein precursor Vtg occurs in follicular cells lining the oocytes. The production of Vtg occurs during early gametogenesis, and it is responsive to estradiol-17β in females, although males also possess the relevant gene, usually unexpressed (Matozzo et al., 2008; Ni et al., 2014), as in fish (Walker et al., 2009). This hormone acts through estradiol receptors, which bind to the promoter region of the Vtg gene (Tran et al., 2016). Long-term exposure to municipal effluents has been found to lead to mussel feminization (development of intersex conditions), with males expressing Vtg transcripts and proteins (Gagné et al., 2011b; André et al., 2020); this also occurs in fish (Jobling and Sumpter, 1995; Fernandez et al., 2007). For example, wild *Elliptio complanata* mussels collected downstream of a municipal effluent plume have been found to consist of 85% females, compared to upstream sites with 42% males. Caging mussels for 1 year (one complete cycle of reproduction) at the site of a municipal effluent dispersion plume has been found to increase the proportion of females from 40% to 70%, with an increase in Vtg-like proteins in both male and female mussels. Induction by estrogens of an increased female-to-male ratio in oysters has been recognized for a long time (Mori et al., 1969), with long-term exposure to estrogens also inducing Vtg in both male and female oysters (Andrew et al., 2010). In *Ruditapes decussatus* clams exposed to a municipal effluent for 30 days, gill tissue extracts revealed estrogenic potential leading to remarkable disruptions in gametogenesis, glycogen content, and Vtg-like proteins (Mezghani-Chaari et al., 2015). Moreover, significant lipid peroxidation (LPO) has been observed in exposed clams, in keeping with the oxidative potential of municipal effluents in both bivalves and fish (Cossu et al., 2000; Gagné et al., 2004 and 2006). However, it is not known whether rainfall overflow containing untreated wastewater significantly contributes to the mussel feminization observed under exposure to treated municipal effluents. A previous study has revealed increased Vtg gene expression at a site downstream of a major municipal effluent and at one of the rainfall overflow sites investigated (André et al., 2020). However, this study also showed that Vtg receptors in the gonad were decreased at the overflow site and that nuclear autoantigenic sperm protein gene expression was elevated in females. The observed responses at the gene expression level should be validated via measurement of actual Vtg protein in gonad tissues.

The purpose of this study, therefore, was to examine the combined effects of sewer overflow and municipal effluents on freshwater *Elliptio complanata* mussels. Mussels were caged at sites releasing combined sewer overflow during heavy rain events and the corresponding treated effluent for 3 months during the summer season, when many storm events occur, altering water levels and quality. Toxicity was examined at levels of increasing complexity, such as the levels of neuroendocrinology, energy reserves, tissue weights, and general health status, in an attempt to understand the modes of action of these urban contaminants from rainfall overflows and from a primary-treated municipal effluent. The objective was to highlight toxicity profiles associated with combined sewer overflow and treated municipal effluents in a representative mussel species of the St. Lawrence River.

## Methods

### Mussel caging experiments

**FIGURE 1:**
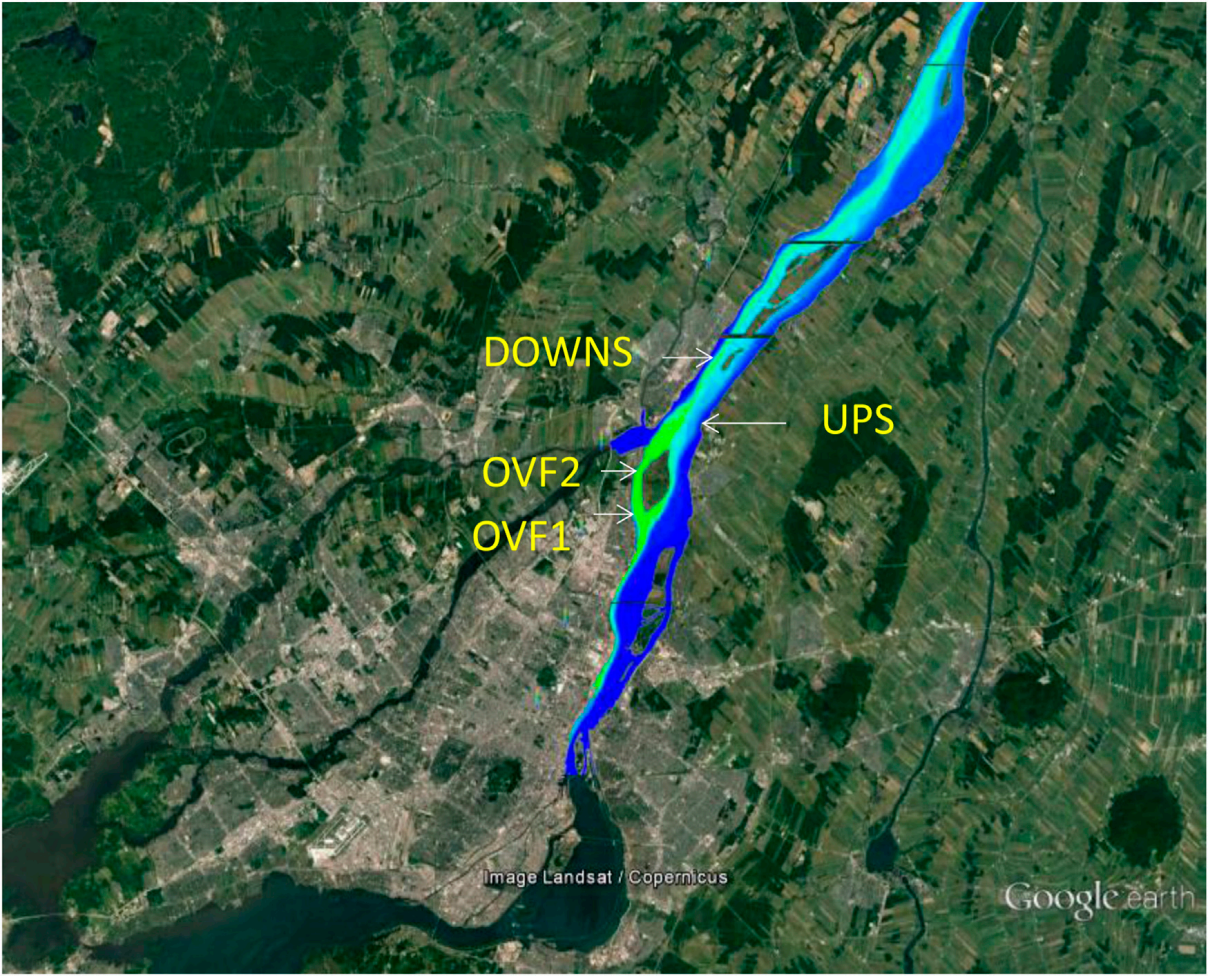
Geographical location of the study sites. The mussel caging sites (OVF1, OVF2, UPS, and DOWNS) are shown. A simulated dispersion plume is shown on the map for the rainfall overflow and downstream sites. The direction of flow of the St. Lawrence River is from the lower left to the upper right of the map. Source: Google Maps.

*Elliptio complanata* mussels (between 150 and 200 individuals of >4 cm shell length) were collected at lakes in the Laurentians (Québec, Canada) under permit from the Wildlife Services of the province of Québec (Canada). The mussels were transported back to the laboratory at 4°C in ice boxes and placed in aquariums (30 mussels per aquarium) containing 60 L of UV-treated and charcoal-filtered tap water from the City of Montréal. They were maintained at 15°C for 20 days before the caging experiment and were fed three times weekly with a commercial phytoplankton (Phytoplex^®^) food mix. The mussels were then placed in cylindrical polyethylene nets (1 m long × 0.5 m diameter; 1 cm diameter mesh) attached to a 1-kg cement block. They were immersed at a depth of at least 1 m at four different sites: two rainfall overflow sites on the north shore of the St. Lawrence River (OVF1: 45°38′26.3″N; 73°29′15.6″W; OVF2: 45°36′05.2″N; 73°30′33.6″W), downstream of the City of Montreal but prior to the municipal effluent discharge (Figure 1); one site downstream of the City of Montreal but upstream (by 2 km) of a major municipal effluent dispersion plume (UPS: 45°39′28,5″N; 73°28′37,9″W); and one site located 8 km downstream of the municipal effluent plume (DOWNS: 45°44′23,9″N; 73°25′43,9″W). This DOWNS site has previously been shown to feminize mussels caged there for 1 year (Blaise et al., 2003). The mussel cages were allowed to stand for 3 months, during the period of July–October of 2017. The cages were inspected twice monthly to check for significant mussel mortality or cage losses. At the end of the exposure period, the mussels were depurated overnight in clean water to permit cleaning and emptying of the gut contents. Surface water samples (4 L) were collected at the beginning of the experiment, at 1.5 months, and at the time of cage retrieval to test for basic water quality measures (pH, conductivity, total suspended solids, dissolved organic carbon, and ammonia levels) and pharmaceuticals via high-performance liquid chromatography–mass spectrometry (Boisvert et al., 2012; Darwano et al., 2014; AWWA, 2017). The following compounds were measured in surface waters and mussels: methotrexate, carbamazepine, caffeine, acebutolol, venlafaxine, atrazine (and its metabolite desehtylatrazine), sulfamethoxazole, diclofenac, and ibuprofen. Another group of mussels (10) was set aside for administration of the stress-on-stress test (air survival) as a general indicator of mussel health (André et al., 2020). Briefly, mussels from each of the four sites were placed in plastic vessels and kept at 80% humidity at 20°C in an incubator. Mussel weights were measured each day, and death was judged on shell opening after handling. The time to death was expressed in the form of days, and the percentage weight loss was determined as follows: 100 × (starting weight beginning–weight at death/starting weight).

### Biomarker analyses

For this analysis, 12 mussels per site were randomly selected and weighed, and the digestive gland and gonad were dissected on ice. The condition factor (CF: mussel weight/shell length) and the soft tissue weight ratio (SFT: soft tissue wet weight/total mussel weight) were determined. The digestive gland and gonad were dissected to determine the digestive gland index (DGI: digestive gland wet weight/soft tissue wet weight) and the gonadosomatic index (GSI: gonad wet weight/soft tissue wet weight) and stored immediately at −85°C. The tissues (digestive gland and gonad) were thawed on ice for 30 min. Subsequently, 4 volumes of 100 mM NaCl containing 10 mM Tris-acetate, pH 8, 1 mM EDTA, 1 mM KH2PO4, 0.1 mM dithiothreitol, and 1 μg/mL aprotinin were added and homogenized using a Teflon pestle tissue grinder (4–5 passes), and a portion was centrifuged at 12,000 × g for 30 min at 2°C. The supernatant (S12 fraction) was collected for evaluation of acetylcholinesterase (AChE), serotonin (Ser), Dop, metallothionein (MT), glutathione S-transferase (GST), and arachidonate-dependent cyclooxygenase (COX). The samples were stored at −85°C until analysis.

Energy reserves in the gonad homogenates were determined by measurement of total proteins, sugars, and lipids. For total proteins, the homogenate was diluted 1/20 in 10 mM NaOH and incubated for 1 h at room temperature to allow protein denaturation. Subsequently, 5 µL of the dilution was tested for proteins using the protein-dye potential and bovine serum albumin for calibration (Bradford, 1976). These data are expressed in the form mg proteins/g gonad or digestive gland, with values normalized against the upstream site, which was the reference site in the present field study. Total sugars were determined in the gonad according to the microplate anthrone reaction (Jermyn, 1975), using glucose for calibration. These data are expressed in the form μg sugars/g gonad. Finally, total lipids in the gonad homogenate were determined using the microplate phosphovanilin reaction (Frings et al., 1972). Standard solutions of Triton X-100 were used for calibration. These data are expressed in the form μg lipid equivalent/gonad weight. Mussels (4 per site) were also set aside for the determination of total heterotrophic counts (before freezing) following standard methods (AWWA, 2017). The soft tissues were homogenized in one volume of sterile bidistilled water and allowed to stand for 30 min. Serial dilutions of the homogenate were added to agar plates for assessment of total heterotrophic bacteria counts.

Lipid peroxidation (LPO) was determined in the digestive gland and gonad homogenates according to the thiobarbituric acid reactant methodology (Wills, 1987). Aldehyde reactants were measured by fluorescence at 540 nm excitation and 600 nm emission (Synergy-4, BioTek Instruments, United States). Tetramethoxypropane standards (a stabilized form of malonaldehyde) were used for calibration. These data are expressed in the form μg thiobarbituric acid reactants/mg proteins. DNA strand break levels were determined in the homogenates via DNA precipitation assay using fluorescent detection of DNA strand breaks with the Hoechst dye (Olive, 1988; Bester et al., 1994). Briefly, the homogenate was treated with SDS in alkaline media (pH 11–12) and centrifuged in the presence of KCl to assist in the precipitation of genomic DNA. Strand breaks remaining in solution were determined with Hoescht dye at 350 nm excitation and 450 nm emission (Synergy-4, BioTek Instrument, United States). These data are expressed in the form µg DNA strand breaks/mg proteins in the gonad or digestive gland, with values normalized against the upstream site. Dihydrofolate reductase (DHFR) activity was also determined in gonad tissues as a marker of DNA synthesis, as previously described (Gagné et al., 2011a). The assay was performed on the S12 fraction in the presence of NADPH and dihydrofolate (50 µM each). These data are expressed in the form of the decrease in relative fluorescence units (NADPH)/min/mg protein.

Levels of metallothioneins (MT) were determined using the silver saturation assay (Scheuhammer and Cherian, 1986; Gagné, 2014). Briefly, the S12 fraction was mixed with 1 ppm Ag+ in 100 mM glycine-NaOH, pH 8.5, and the excess silver was removed by two additions of hemoglobin (2% w/v), heat denaturation at 100°C for 2 min, and centrifugation at 10,000 × g for 2 min. The remaining silver in the supernatant was determined via graphite furnace atomic absorption spectrometry with silver nitrate standards, 1% ammonium phosphate (matrix modifier), and rabbit MT for validation. These data are expressed in the form of μg MT equivalents/mg proteins, with values normalized against the upstream site. Activity in 7-ethoxyresorufin O-deethylase (EROD) was determined in the S12 fraction, as described previously (de Lafontaine et al., 2000). Briefly, the S12 fraction (50 μL) was mixed with 150 μL of 10 μM 7-ethoxyresorufin and 100 μM NADPH in 50 mM KH_2_PO_4_, pH 7.4, for 45 min. The appearance of 7-hydroxyresorufin was determined by fluorescence at 540 nm excitation and 600 nm emission using a microplate reader (Synergy-4, BioTek Instruments, United States). These data are expressed in the form of increased relative fluorescence units/min/mg proteins.

COX activity and GST activity were also determined in the S12 fraction in 96-well microplates, as described elsewhere (Gagné et al., 2015). Enzyme activity was expressed in the form of change in substrate/min/mg proteins in the gonad or the digestive gland for COX and GST activity, respectively. For AChE, activity was determined using the acetylthiocholine substrate and Ellman’s reagent, as described in Dellali et al. (2001). These data are expressed in the form of change in absorbance (412 nm)/min/mg proteins. Finally, Dop and Ser levels were determined using an enzyme-immunoassay methodology (Gagné et al., 2011). These data are expressed in the form ng serotonin or dopamine/mg proteins in the S12 fraction.

### Western blot immunoanalysis of *Elliptio complanata* Vtg

A small peptide was synthetized (Thermo Fisher Scientific) from the Vtg protein sequence (position 2296:2313; RKLLQDSKFRPLDELKHK) corresponding to the vitellogenin gene in the RNA seq *de novo* transcriptome experiment as described in André and Gagné (2020). This synthetic peptide served as an antigen for the production of custom VTG antibodies (Thermo Fisher Scientific, IL, United States). For mussel gonad homogenates, tissues were centrifuged at 15,000 × g for 20 min at 4°C. The resulting supernatants (S15) were collected, and protein concentration was determined as described above. The S15 samples were mixed with an appropriate volume of 4× Laemmli sample buffer and DTT (Bio-Rad, Mississauga, ON), heated at 70°C for 10 min, and stored at −20°C until analysis. Proteins (20 μg) were separated using high-resolution 4%–15% SDS-polyacrylamide pre-casted gels (Criterion, Bio-Rad, Mississauga, ON) followed by electro-transfer onto low-fluorescence polyvinylidene difluoride membranes (0.45 µm) using the Trans-Blot Turbo Transfer System (Bio-Rad, Mississauga, ON). Transfer efficiency was assessed using the “Protein Gels” and “Stain-Free Gels” applications of the ChemiDoc Imaging System with an activation time of 45 s and “Rapid Auto Expose” settings. Membranes were blocked for 1 h in PBS containing 1% powdered skim milk and 0.05% Tween-20 and then incubated for 3 h with antiserum against *Elliptio complanata* VTG (diluted 1/500 in the blocking solution). After three washing steps with Tris-buffered saline (20 mM tris-HCl, pH 7.6, 150 mM NaCl), horseradish peroxidase-conjugated goat anti-rabbit secondary antibodies (1:1000, Enzo life science, ADI-SAB-300J) were applied and the samples were incubated for 1 h at room temperature. Immunoreactive bands were detected via enhanced chemiluminescence (Clarity™ Western ECL Substrate, Bio-Rad) and imaged using the ChemiDoc Imaging System (Bio-Rad Laboratories). VTG staining at the 250 kDa position (found in females only) was analyzed via densitometry using the Un-Scan-It software (Silk Scientific, Inc., Orem, UT). These data are expressed in the form pixel number/mg proteins in the S15 fraction of the gonad.

### Data analysis

The mussels (*N* = 30 per cage) were selected to have an optimal range of shell lengths between 5 and 8 cm in order to minimize the influence of size on biomarker responses. The data were subjected to rank analysis of variance (ANOVA) followed by the Conovan–Inman test to highlight significant differences relative to the upstream site, which was treated as the reference site in this study. Correlations between variables were assessed via the Pearson product-moment test. Principal component analyses were also performed to determine the most important biomarkers that could identify rainfall overflow and downstream sites specifically based on factorial scores. The significance threshold was set at *p* < 0.05, and all tests were performed using the SYSTAT software package (version 13, United States).

## Results

The basic physicochemical properties of the surface water samples (*N* = 3 grab samples obtained at the time of cage collection) were determined for caged mussels at the upstream (UPS), overflow (OVF1 and OVF2), and downstream (DOWNS) sites (Table 1). The data revealed that pH, conductivity, suspended matter values, and dissolved organic carbon did not vary across the sites. However, total organic carbon and total ammonia contents were higher at the downstream site relative to the upstream site. The rain overflow sites did not show differences relative to the upstream site. The levels of pharmaceuticals commonly found in urban effluents were determined in the surface water samples (Table 2). The data revealed that the downstream site contained higher levels of caffeine, carbamazepine, acebutolol, sulfamethoxazole, venlafaxine, and ibuprofen compared to the upstream site. The following compounds were also increased at the rain overflow sites: caffeine, carbamazepine, atrazine, and ibuprofen. Atrazine levels at the OVF2 site were higher than those detected at the downstream site.

**TABLE 1:** Water quality properties of the caging sites.

	pH	Conductivity µS*cm^−1^	Suspended matter (mg/L)	Total organic carbon (mg/L)	Dissolved organic carbon (mg/L)	Ammonia (mg/L)	Total rain (mm)
UPS	8.4 ± 0.1	283 ± 5	3 ± 1	3 ± 0.2	2.8 ± 0.2	0.004 ± 0.001	164
OVF1, OVF2	8.37 ± 0.06	280 ± 7	17 ± 2	3.1 ± 0.11	2.9 ± 0.1	0.007 ± 0.001	
DOWNS	8.36 ± 0.09	280 ± 5	4 ± 2	3 ± 0.1	2.9 ± 0.2	0.006 ± 0.002	
	8.2 ± 0.1	284 ± 9	4 ± 1	3.5 ± 0.1*	3.2 ± 0.2	0.123 ± 0.015*	

Asterisks indicate significant differences from the upstream site. Data presented are means with standard deviations across measurements taken each month during the 3-month exposure period.

**TABLE 2:** Occurrence of pharmaceutical products in surface waters at the caging site.

Sites	OVF1 (ng/L)	OFV2 (ng/L)	Upstream (ng/L)	Downstream (ng/L)
Caffeine	25.7 ± 0.1	54 ± 0.3*	28.7 ± 0.3	1145 ± 2*
Carbamazepine	2.48 ± 0.03	3.45 ± 0.09*	2.58 ± 0.15	5.37 ± 0.11*
Atrazine	46.4 ± 0.8	91.2 ± 2.9*	46.8 ± 0.5	48.4 ± 2.2
Acebutolol	0.29 ± 0.01	0.45 ± 0.02	0.24 ± 0.002	2.3 ± 0.03*
Sulfamethoxazole	<LOD	<LOD	<LOD	4.41 ± 0.05*
Venlafaxine	1.14 ± 0.01	2.04 ± 0.04	1.02 ± 0.02	6.37 ± 0.016*
Ibuprofen	10.1 ± 0.6*	7 ± 0.31*	8.52 ± 0.42	51.1 ± 2.2*

LOD, limit of detection of the instrument. * indicates significance from the upstream (reference) site.

**FIGURE 2:**
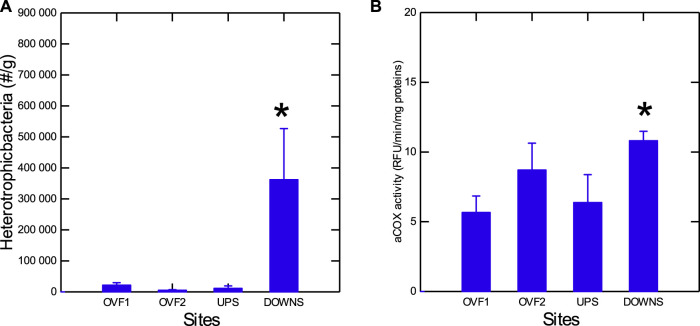
Bacterial loadings and inflammation in mussels exposed to urban effluent and rainfall overflow. Total bacterial counts **(A)** and arachidonate cyclooxygenase **(B)** were determined in mussel tissues following the 3-month exposure period. The data presented are means with standard deviations. *statistically significant difference from the upstream (reference) site.

**FIGURE 3:**
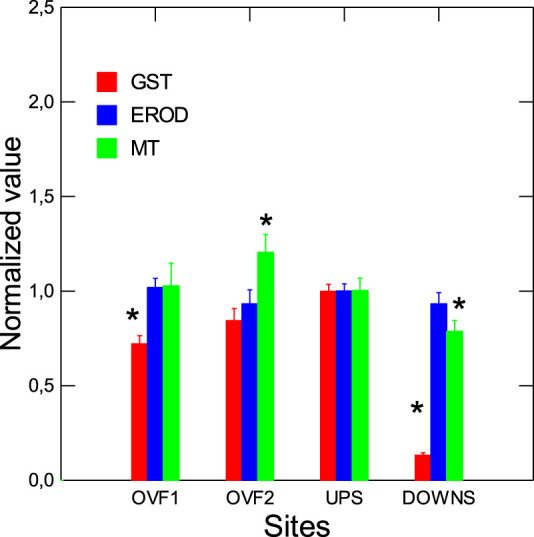
Biotransformation activity in mussels exposed to urban pollution. Xenobiotic biotransformation activity was determined by examining changes in GST, EROD-like activity, and MT. The data presented are means with standard deviations. *statistically significant difference from the upstream (reference) site.

The levels of total heterotrophic bacteria and inflammation (as determined by arachidonate cyclooxygenase or COX) were evaluated in mussel tissues (Figure 2). These measurements were made to confirm that caged mussels at the downstream site were indeed exposed to the effluent dispersion plume, since the municipal effluent of Montréal does not support a disinfection step (a new disinfection step using ozone is to be implemented in 2024–25). Total bacterial loadings and COX activity were significantly higher in mussels at the downstream site compared to those at the reference site. Mussels at the overflow sites did not exhibit any difference in bacterial loadings or COX activity. Morphological changes in caged mussels were examined in terms of the condition factor (CF: mussel weight/shell length), soft tissue weight ratio, GSI, and DGI (Table 3). Moreover, air survival time (corrected to weight loss) was used as a measure of health status. CF, GSI, and DGI were not significantly altered in mussels caged at the two overflow sites or at the downstream site relative to the upstream site. The soft tissue ratio was significantly increased at the downstream site and was significantly correlated with total bacteria tissue loadings (*r* = 0.88). Biotransformation activity was determined by GST, EROD-like activity, and MT as a measure of heavy metal exposure (Figure 3). GST and MT levels were decreased at the downstream site compared to the upstream site. MT levels were increased at the OVF2 site, and GST was decreased at the OVF1 site. Correlation analysis revealed that MT levels were significantly correlated with CF (*r* = 0.87), bacterial loadings (*r* = −0.56), and COX (*r* = −0.47) (Supplementary Table S1).

**TABLE 3:** Morphological characteristics of caged mussels.

Site	CF	SFT	GSI	DGI	Lethality time (days)
OVF1	0.57 0.04	0.15 ± 0.01	0.23 ± 0.05	0.144 ± 0.02	11 ± 4
OVF2	0.55 0.02	0.18 ± 0.005	0.155 ± 0.007	0.121 ± 0.007	7 ± 1.3
UPS	0.53 0.03	0.17 ± 0.007	0.16 ± 0.01	0.11 ± 0.004	9.3 ± 2
DOWNS	0.54 0.03	0.20 ± 0.01*	0.27 ± 0.04	0.14 ± 0.01	8.4 ± 2

CF, condition factor; SFT, soft tissue weight ratio; GSI, gonadosomatic index; DGI, digestive gland index. *statistically significantly different from the upstream site

**FIGURE 4:**
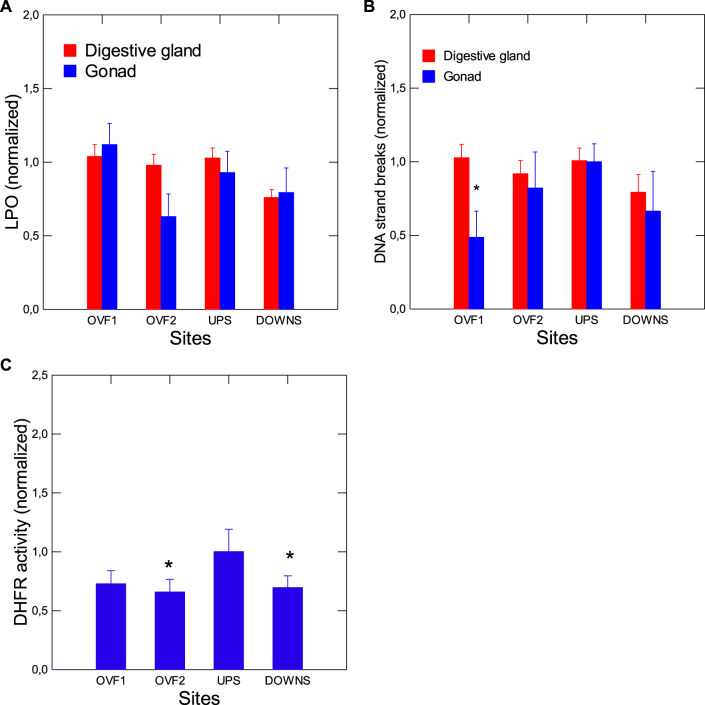
Tissue damage in mussels exposed to wastewater and overflow. Tissue damage was determined by examining changes in the digestive gland and gonad in LPO **(A)**, DNA strand breaks **(B)**, and DHFR activity (purine synthesis) **(C)**. The data presented are means with standard deviations. *statistically significant difference from the upstream (reference) site.

Tissue damage in both the digestive glands and the gonads of caged mussels was determined by evaluating changes in LPO, DNA strand breaks and DHFR as a measure of purine metabolism (Figure 4). No changes in LPO or DNA strand break levels were found in mussels caged at the downstream or overflow sites relative to the upstream site, in either the digestive gland or the gonad tissues. However, DNA strand breaks in the gonad were decreased at one of the overflow sites (OVF1). DHFR activity was significantly decreased at the downstream site and at one overflow site. Additionally, LPO in the digestive gland was correlated with the GSI (*r* = −0.68), bacterial counts (*r* = −0.62), and SFT (*r* = −0.6). There was a significant increase in residual LPO levels in the digestive gland when this was corrected for bacterial counts in tissues at the downstream and the 2 overflow sites as compared to the upstream site (suggesting that high bacterial counts decrease LPO, as with COX activity). LPO in the gonad was significantly correlated with DNA strand breaks in the digestive gland (*r* = −0.56). In contrast, DNA strand breaks in the digestive gland were correlated with CF (*r* = 0.65), GSI (*r* = −0.57), gonad DNA breaks (*r* = 0.77), EROD activity (*r* = 0.79), COX activity (*r* = −0.57), bacterial counts (*r* = −0.74), and MT (*r* = 0.71). Gonad DNA strand breaks were significantly correlated with gonad LPO (*r* = −0.57) and EROD activity (r = 0.55). The activity of DHFR, as a measure of the production of purine precursors for DNA synthesis, was significantly correlated with LPO in the digestive gland (*r* = 0.53) and gonad (*r* = 0.59), which was decreased at the downstream site, although the difference was not significant for one of the overflow sites (OVF1).

**FIGURE 5:**
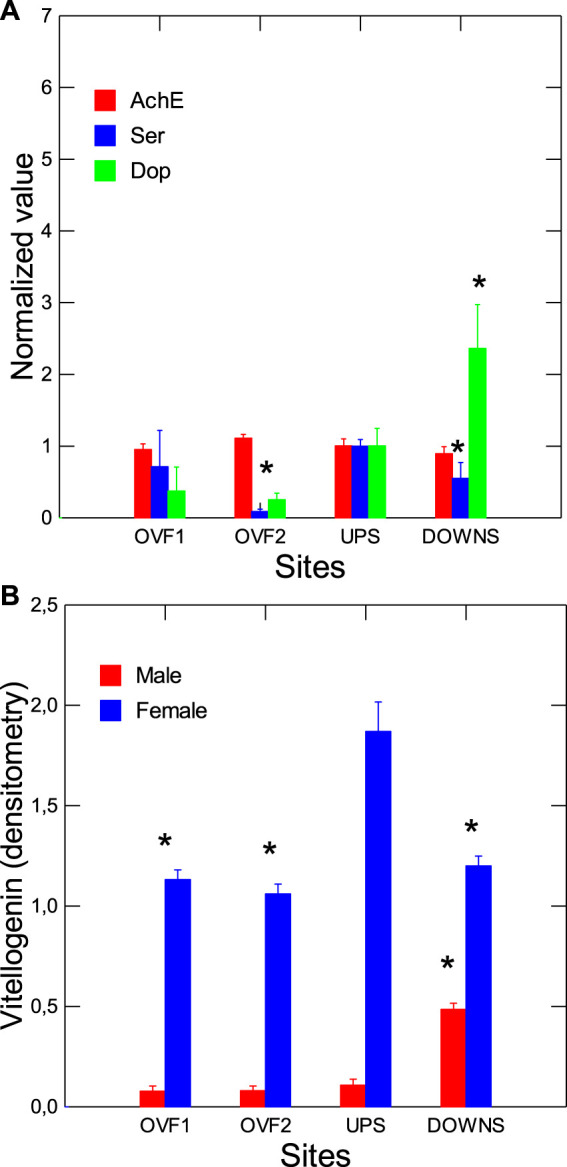
Neuroendocrine status of caged mussels at rain overflow and downstream sites. The neuroendocrine status was examined in terms of changes in gonad AChE activity, Ser, Dop **(A)**, and Vtg levels **(B)**. The data presented are means with standard deviations. *statistically significant difference from the upstream (reference) site.

**FIGURE 6:**
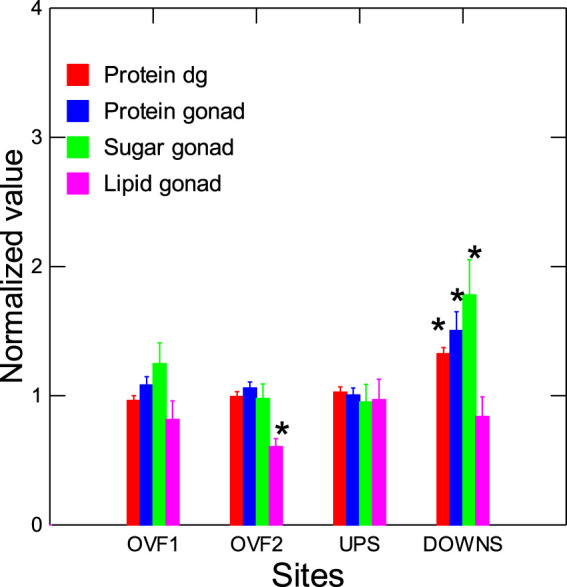
Energy status in mussels exposed to overflow and at the site downstream of the effluent. Energy status was characterized by measuring changes in total proteins, sugars, and lipids. The data presented are means with standard deviations. *statistically significant difference from the upstream (reference) site.

The neuroendocrine status of mussels was studied via the gonad tissues (Figure 5). A decrease in Ser and an increase in Dop were observed at the downstream site (Figure 5A). Levels of Vtg, as determined by western blot analysis using raised antibodies for *Elliptio complanata*, were determined in males and females. At the downstream site, these levels were significantly increased in males, with a decrease in female mussels (Figure 5B). In the correlation analysis, male Vtg was correlated with CF (*r* = −0.68), GSI (r = 88), SFT (*r* = 0.92), digestive gland DNA breaks (*r* = −0.65), and MT levels (*r* = −0.55). Vtg levels were lower in females at the overflow sites, but no changes were found in males. Correlation analysis revealed that Dop levels were significantly correlated with COX (*r* = 0.6), GST (*r* = 0.68), male VTg (*r* = 0.59), and gonad LPO (*r* = 0.61). Vtg in females was significantly correlated with Ser levels (*r* = 0.61), AChE (*r* = −0.6), and DGI (*r* = −0.68) (Supplementary Table S1). Mussel energy status was examined by evaluating changes in total levels of proteins, sugars, and lipids (Figure 6). The data revealed that total proteins in both the digestive gland and the gonad and total sugars were significantly increased at the downstream site. Total lipids were reduced at one of the overflow sites. Correlation analysis revealed that total lipid levels were significantly correlated with AChE (*r* = −0.73), while total sugars were correlated with CF (*r* = −0.55) and EROD (*r* = −0.59).

**FIGURE 7:**
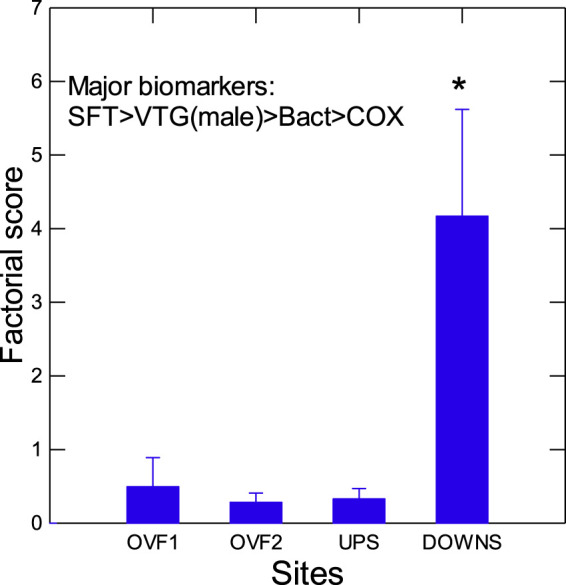
Principal component analysis of biomarker responses in caged mussels. Principal component analysis was performed to extract the main effects related to various sites. The data presented are the mean and standard deviation of the factorial score at each site and the most important biomarkers related to each score.

In an attempt to obtain a global perspective on biomarker responses in mussels placed at rainfall overflow and municipal dispersion plume sites, a principal component analysis was performed for one factor in order to extract the most important biomarkers (Figure 7) for these effects. The factorial scores, explaining 45% of the variance, were significantly higher at the downstream site compared to the upstream and overflow sites, suggesting that the municipal effluent plume was the main driver of effects in this system and that the contribution of the overflow sites was negligible in terms of difference from the upstream site. The factorial scores were mainly explained by SFT, elevation of VTG in male mussels, bacterial loadings, and inflammation (COX), all of which were significantly increased at the downstream site of the municipal effluent dispersion plume.

## Discussion

Caged mussels exposed to an un-disinfected municipal effluent plume 8 km downstream of the point of discharge accumulated significant amounts of microorganisms and other suspended material in addition to dissolved contaminants. The levels of heterotrophic bacteria in mussel tissues could serve as a simple means of measuring exposure to suspended particles from municipal effluents, provided that bacterial growth is controlled by the immune system. The accumulation of bacteria in tissues was associated with increased SFT, inflammation, and Vtg proteins in males. Moreover, soft tissue weight ratio was significantly increased at the downstream site only, which suggests that the mussels were feeding on the suspended matter released by the municipal effluent. An increase in soft tissues was previously reported in mussels and clams exposed to municipal effluent in the laboratory (Nobles and Zhang, 2015). In another study, mussel caging was used to determine the ecotoxicological impacts of leachates from an open dump site (Yusseppone et al., 2020). Mussels were caged for over 30 days at these sites and showed signs of stress in the form of lysosome membrane destabilization in the hemocytes; additionally, certain tissues (gills and adductor muscles) exhibited elevated copper and iron levels. Moreover, there was a reduction in condition factor and in the digestive gland index. Growth was also increased under exposure to municipal effluents. Increased mussel size, condition factor, and GSI have also been observed in wild mussels exposed to two consecutive municipal effluents in a Canadian river (Gagné et al., 2011). In the present study, iflammation was determined by examining changes in arachidonate COX, and the results corroborated earlier findings on the induction of COX activity by a physicochemically treated effluent (Gagné et al., 2005). In another study, mussels challenged with the pathogen *Vibrio anguillarum* during exposure to the same municipal effluent that provided the setting for this study were found to exhibit increased COX and GST activity (Gagné et al., 2015). This supports the case that microorganisms contribute to inflammation responses in mussels under exposure to municipal effluent. Caging of mussels at the overflow sites did not lead to the accumulation of bacteria or to inflammation in the digestive tissues. However, COX activity is also involved in spawning activity, along with Ser levels in tissues (Ford and Fong, 2016). In the present study, Ser levels were decreased in mussels caged at the downstream site, and COX activity was not correlated with Ser levels in mussels. This suggests that COX activity was principally driven by inflammation and was not involved in spawning. Although no important changes in DNA strand breaks were observed in the present study, a significant decrease in gonad DNA strand breaks was observed at one overflow site, and a non-significant decrease in DNA breaks was observed at the downstream site. Decreased DHFR activity was also observed at the downstream site, suggesting a decrease in DNA synthesis activity. The amount of DNA strand breaks has been shown to be a function of the formation of DNA adducts, or modifications such as an increase in the number of alkali-labile sites (abasic sites) and the rate of DNA repair activity, which consists of the excision of large DNA strands and the replacement of nucleic bases. In organisms exposed to genotoxic compounds, the levels of DNA strand breaks could increase initially, as strand breaks are formed during repair, but this response could be followed by a gradual decrease in DNA strand breaks as the repair mechanism becomes saturated and depleted. A decrease in DNA strand breaks suggests that decreased DNA repair activity is associated with the accumulation of DNA damage at the cytogenetic level, as previously observed (Gagné et al., 2011a; Debenest et al., 2012). Thus, a decrease in gonad DNA strand breaks could indicate cytogenetic damage that could prove harmful to mussel offspring, as has been shown in invertebrates (Lacaze et al., 2011).

In this study, increased levels of SFT and GSI were associated with the induction of Vtg in males and were also correlated with bacteria counts in tissues. The increased expression of Vtg proteins in males is a well-known marker of feminization by compounds acting as endocrine disrupters. Municipal effluents have been recognized to feminize mussels by increasing the female/male ratio, with males producing more Vtg in *Elliptio complanata* (Blaise et al., 2003; Gagné et al., 2011) and in oysters (Andrew-Priestly et al., 2012). Indeed, the sex ratio in mussels caged in special benthic pens for over 1 year (covering one reproductive cycle) in a municipal effluent dispersion plume increased from 41% to 61% females. Additionally, Vtg-like proteins were elevated based on high-resolution gel electrophoresis and silver staining and alkali-labile phosphates (Blaise et al., 2003). In another study, wild *Elliptio complanata* mussels collected at two sites downstream of municipal discharges were found to be strongly feminized, with the proportion of females reaching 85% of the population, as compared to 45% males at upstream sites (Gagné et al., 2011). Male mussels also contain significant amounts of Vtg proteins, which are correlated with Dop levels, whose levels are increased during early gametogenesis (Osada and Nomura, 1989; Gagné et al., 2011). In a recent study, gene expression in Vtg and its receptors (which was higher in females than in males) was found to be increased in male *Elliptio complanata* mussels exposed for 3 months to the same municipal effluent (André and Gagné, 2020), corroborating the observed increase in Vtg protein as determined by western blotting using raised antibodies in the present study. The decrease in Vtg in females is consistent with the expression of the nuclear autoantigenic sperm protein gene in females. This could suggest a form of “masculinization” effect on female gonads. The masculinization properties of municipal effluents are much more poorly understood, but some evidence suggests that these effluents could also produce androgenic effects. For example, levels of androgen receptors have been found to be increased in mosquitofish (females > males) in rivers impacted by municipal wastewater (Huang et al., 2016). The increased expression of androgen receptors in females was found to be associated with an increased gonopodium-like anal fin in females, reaching half the length found in males. For mussels, the present study is the first indication of androgenic effects in females based on decreased Vtg proteins in gonads in mussels at the downstream site, along with the previously reported increase in sperm protein gene expression in caged mussels exposed to the same effluent (André et al., 2020). There has been considerable debate as to whether estrogens, which abound in municipal effluents, could increase Vtg gene expression and proteins in freshwater and marine mussels. In some cases, Vtg induction has been found to be stimulated by estradiol-17β (Andrew et al., 2010; Ni et al., 2014; Tran et al., 2016; Leonard et al., 2017). In other cases, estradiol-17β has failed to induce Vtg in mussels (Won et al., 2005; Puinean et al., 2006; Fernández-González et al., 2021). These results have been followed by the identification of confounding variables, such as dosage, route of exposure (injection vs. water), sexual differentiation stage (protandry is observed in some unionids), period of gametogenesis (early vs. late) in mussels, specificity of the alkali-labile phosphate method (variable phosphorylation of Vtg in invertebrates), age, and high steroid esterification potential (inactivation) in bivalves. Nevertheless, it appears that bivalves express an estrogen receptor ortholog, which is constitutively expressed in oysters (Tran et al., 2017) and increased by exposure to estradiol-17β (Tran et al., 2017). Recent studies have tended to confirm that mussels respond to estrogenic compounds, since they express an estrogen receptor ortholog to vertebrate receptors, but they are seemingly less responsive than fish to estradiol (Tran et al., 2016; 2017). The estrogen receptor ortholog was recently identified in bivalves capable of activation of estrogen-responsive elements by exposure to estradiol-17β via a transfected yeast system (Liu et al., 2023). This study also showed that bivalve estrogen receptors were less potent than fish estrogen receptors in activating the estrogen receptor–responsive elements for Vtg and other genes. From the perspective of environmental protection, the induction of Vtg in males is a useful marker of feminization, and recent evidence supports the hypothesis that estrogenic compounds (able to interact with the estrogen receptor ortholog) contribute, at least in part, to the feminizing effects of municipal effluents. The ultimate goal, from this perspective, is to optimize wastewater treatment systems to remove this effect for the protection of sessile bivalve populations. In a pilot study, the addition of an ozonation step to a primary treatment step significantly reduced the induction of Vtg-like proteins in mussels in addition to reducing microorganisms and viruses (Gagné et al., 2009).

In conclusion, this study sought to compare the biomarker profile of rain overflows (which are expected to increase with climate change) and a municipal dispersion plume in caged mussels at specific sites in the St. Lawrence River. Based on the observed responses, primary-treated effluent has more impact than rainfall overflow sites on mussels. In addition, mussels caged at the downstream site readily underwent changes in terms of Vtg proteins in males (a marker of feminization), increased inflammation (COX), and increased soft tissue mass. Caging at the rainfall overflow sites had no effects on these markers, although reduced Vtg levels in females were observed at one overflow site. Other effects specific to rainfall overflow sites were increased MT and decreased lipids, Dop, and DNA strand breaks. This suggests that rainfall overflow does not have the same effects as primary-treated effluent, and the latter is responsible for the majority of the feminizing effects seen in mussels. This study design will nevertheless permit investigation of the contribution of wastewater overflow following rain events in terms of intensity and frequency and that of upgrades in wastewater treatment processes.

## Data Availability

The original contributions presented in the study are included in the article/Supplementary Material; further inquiries can be directed to the corresponding author.
