# Supplementary material for: Comparative toxicity of urban wastewater and rainfall overflow in caged freshwater mussel Elliptio complanata
Source: Front Physiol. 2023 Aug 10;14:1233659. doi: 10.3389/fphys.2023.1233659 (PMC10449329; doi:10.3389/fphys.2023.1233659)
Supplement: Supplementary file 1 [file Table1.docx]

**Table 1S. Correlation analysis of biomarkers**

**Pearson Correlation Matrix**

¦ Bact # CF GSI SFT DGI WL GST LPOdg LPOgon DNAdg DNAgon

-------------------------------------------------------------------------------------------------------------------

Bact# ¦ 1

CF ¦ **-0.6** 1

GSI ¦ **0.84** **-0.59** 1

SFT ¦ **0.88** **-0.80** **0.93** 1

DGI ¦ 0.01 0.18 0.15 0.1 1

WL ¦ **-0.64 0.61 -0.63 -0.64** 0.53 1

GST ¦ 0.13 -0.01 0.17 0.13 0.04 -0.37 1

LPOdg ¦ **-0.62** 0.29 **-0.68** **-0.60** -0.03 **0.59** 0.1 1

LPOgon ¦ 0,08 -0.43 0.17 0.25 0.28 -0.26 **0.55** 0.1 1

DNAdg ¦ **-0.74** **0.65** **-0.57** **-0.64** -0.03 0.54 -0.11 0.36 -0.48 1

DNAgon ¦ -0.25 0.26 -0.01 -0.08 -0.16 0.06 -0.08 -0.11 **-0.57** **0.77** 1

EROD ¦ -0.46  **0.57** -0.29 -0.43 0.12 0.39 0.05 0.2 -0.3 **0.79** 0.55

SUG ¦ 0.10 -0.55 0.4 0.37 0.01 -0.33 0.07 -0.1 0.5 -0.50 -0.30

VTGmale ¦ **0.84 -0.68** **0.88** **0.92** 0.18 **-0.65** 0.49 -0.46 0.45  **-0.65** -0.19

VTGfem ¦ -0.19 -0.12 -0.23 -0.15 **-0.68** -0.35 0.08 0.12 0.03 0.30 0.27

LIP ¦ 0.09 0.27 0.27 0.05 0.19 -0.24 0.43 -0.23 0.32 0.02 0.02

AChE ¦ -0.05 0.00 -0.16 -0.03 0.44 **0.65** **-0.57** 0.17 -0.36 0.05 -0.04

COX ¦ 0.31 -0.09 -0.02 0.00 -0.35 -0.34 0.39 0.24 0.29 **-0.57** **-0.68**

DHFR ¦ -0.13 -0.46 0.04 0.20 0.28 0.23 0.05 0.53 0.49 -0.06 -0.12

Ser ¦ -0.33 0.14 -0.44 -0.4 -0.08 0.1 0.08 0.30 0.41 0.1 -0.34

Dop ¦ 0.41 -0.42 0.23 0.34 -0.11 -0.45 **0.68** 0.12 **0.61** -0.42 -0.43

MT ¦ **-0.56** **0.87** -0.41 **-0.62** 0.46 **0.71** -0.12 0.15 -0.42 **0.71** 0.38

**-----------------------------------------------------------------------------------------------------------------------**

**Pearson Correlation Matrix (Contd.)**

¦ EROD SUG Vtgmale Vtgfem LIP ACHE COX DHFR Ser Dop MT

SUG ¦ **-0.59** 1

VTGmale ¦ -0.35 0.34 1

VTGfen ¦ 0.36 -0.29 -0.17 1

LIP ¦ 0.37 -0.11 0.22 0.22 1

AChE ¦ -0.11 -0.09 -0.2 **-0.6** **-0.73** 1

COX ¦ -0.41 0.01 0.2 0.11 0.14 -0.37 1

DHFR ¦ -0.15 0.45 0.2 -0.06 -0.24 0.27 -0.13 1

Ser ¦ 0.37 -0.30 -0.29 **0.61** 0.42 -0.40 0.28 0.02 1

Dop ¦ -0.05 -0.05 **0.59** 0.34 0.25 -0.41 **0.6**  0.2 0.43 1

MT ¦ **0.74** -0.48 **-0.55** -0.24 0.22 0.23 -0.47 -0.30 0.06 -0.48 1

--------------------------------------------------------------------------------------------------------------------------------------------------------------------------------------------------

Significant correlations are in **bold** (r≥0.55; p≤0.05). LPOdg: digestive gland LPO; LPOgon: gonad LPO. DNAdg: digestive gland DNA strand breaks; DNAgon: gonad DNA strand breaks.
